# Sex-Dimorphic Interactions of *MAOA* Genotype and Child Maltreatment Predispose College Students to Polysubstance Use

**DOI:** 10.3389/fgene.2019.01314

**Published:** 2020-01-17

**Authors:** Paula J. Fite, Shaquanna Brown, Waheeda A. Hossain, Ann Manzardo, Merlin G. Butler, Marco Bortolato

**Affiliations:** ^1^ Consortium for Translational Research on Aggression and Drug Abuse (ConTRADA), University of Kansas, Lawrence, KS, United States; ^2^ Clinical Child Psychology Program, University of Kansas, Lawrence, KS, United States; ^3^ Departments of Psychiatry and Behavioral Sciences and Pediatrics, University of Kansas Medical Center, Kansas City, KS, United States; ^4^ Department of Pharmacology and Toxicology, University of Utah, Salt Lake City, UT, United States

**Keywords:** polysubstance use, MAOA, child maltreatment, sex differences, gene × environment interactions

## Abstract

Polysubstance use (PSU) is highly prevalent among college students. Recent evidence indicates that PSU is based on gene x environment (G×E) interactions, yet the specific biosocial factors underlying this problem remain elusive. We recently reported that lifetime use of tobacco and cannabis in college students is influenced by the interaction of the X-linked *MAOA* (monoamine oxidase A) gene and child maltreatment. Building on these premises, here we evaluated whether the same G×E interaction may also predict PSU in this population. Students of a large Midwestern university (n = 470; 50.9% females) took part in a computer survey for substance use, as well as childhood trauma exposure, using the Child Trauma Questionnaire (CTQ). DNA was extracted from their saliva samples and genotyped for *MAOA* variable-number of tandem repeat (VNTR) variants. Findings indicated that the highest number of substances were used by male students harboring low-activity *MAOA* alleles with a history of childhood emotional abuse. In contrast, female homozygous high-activity *MAOA* carriers with a history of emotional and physical abuse reported consumption of the greatest number of substances. Our results indicate that PSU among college students is influenced by the interaction of *MAOA* and child maltreatment in a sex-specific fashion. Further studies are warranted to understand the mechanisms of sex differences in the biosocial interplays underlying PSU in this at-risk group.

## Introduction

Polysubstance use (PSU) is a major health concern that has garnered much attention from clinicians and researchers, due to its robust association with substance use disorders and other negative outcomes throughout the lifespan (McCabe et al., 2006; Trenz et al., 2012; Moss et al., 2014). Recent surveys have ascertained that PSU risk is particularly high among college students (Gledhill-Hoyt et al., 2000; Johnston et al., 2004; Mohler-Kuo et al., 2003; Barrett et al., 2006; National Center on Addiction and Substance Abuse at Columbia University, 2007; O'Grady et al., 2008) with alcohol, tobacco, and cannabis being the three most widely used substances in this population (Lipari and Jean-Francois, 2016). Indeed, these drugs share similar trajectories of use among emerging adults, with high rates of comorbidity (Jackson et al., 2008) and simultaneous consumption (Martin et al., 1992; Baggio et al., 2014).

Vulnerability to PSU, and more generally to substance use disorders and related behavioral phenotypes (including externalizing psychopathology), is strongly influenced by both genetic (Uhl et al., 2001; Dick et al., 2009) and environmental factors. Several genes implicated in the predisposition to substance use disorders have been shown to be related to monoamine neurotransmitters, such as serotonin, dopamine, and norepinephrine (Guo et al., 2007; Ducci and Goldman, 2012); these molecules are known to serve a pivotal role in the pathophysiology of drug abuse (Volkow et al., 2007; Fitzgerald, 2013; Müller and Homberg, 2015). Early-life adversity, and particularly child maltreatment is another well-known variable associated with high risk of PSU (Galaif et al., 2001; Leeb et al., 2008; Goldstein et al., 2013; Cohen et al., 2017). It has been estimated that ~70% of adolescents receiving substance abuse treatment have a history of trauma (Funk et al., 2003), and that maltreated children are 300% more likely to develop substance abuse (Kilpatrick et al., 2003). According to recent conceptual frameworks, the pathogenic influence of child maltreatment and other forms of early stress on PSU is moderated by genetic factors (Vink, 2016). However, only limited data are available on the specific interactions of heritable factors and child maltreatment with respect to PSU predisposition.

We recently showed that, among college students, tobacco and cannabis consumption is influenced by the interaction of child maltreatment and the gene *MAOA*, the X-linked gene encoding for monoamine oxidase A (Fite et al., 2018). In line with our report, Stogner and Gibson (2013) also documented that the interplay of this gene with lifetime stress increases the risk for initiation to alcohol and cannabis use in male adolescents. Monoamine oxidase A catalyzes the degradation of serotonin, norepinephrine and dopamine (Bortolato et al., 2008). The best-characterized *MAOA* functional polymorphism is a 30-bp variable number tandem repeat located in its promoter region (*uVNTR*) (Sabol et al., 1998). The six alleles of this genotype feature different numbers of repeats (2, 3, 3.5, 4, 5, and 6) (Huang et al., 2004), in association with different transcriptional efficiency and enzyme activity. The two- and three-repeat variants, which are associated with low activity (Sabol et al., 1998; Deckert et al., 1998; Denney et al., 1999), confer a greater risk for externalizing psychopathology in male carriers with a history of maltreatment (Caspi et al., 2002; Kim-Cohen et al., 2006; Williams et al., 2009; Fergusson et al., 2011).

A large body of evidence has documented that *MAOA uVNTR* variants exert a sex-dimorphic influence on the overall risk and specific clinical manifestations of alcohol use disorders, both *per se* and in interaction with early-life adversity (Samochowiec et al., 1999; Schmidt et al., 2000; Vanyukov et al., 2004; Guindalini et al., 2005; Herman et al., 2005; Ducci et al., 2008; Nilsson et al., 2011). Low-activity *uVNTR* alleles (hereafter designated as *MAOA-L*), for example, are associated with a younger age of onset of alcohol dependence (Vanyukov et al., 1995; Vanyukov et al., 2004) and antisocial alcoholism (Samochowiec et al., 1999) in males. A history of maltreatment predisposes female carriers of high-activity alleles (*MAOA-H*) or male MAOA-L carriers to a greater risk of alcohol use (Nilsson et al., 2011). In alignment with these findings, we found that greater lifetime tobacco use was predicted by the interaction of childhood maltreatment and *MAOA-L* variants in males and *MAOA-H* alleles in females (Fite et al., 2018).

Given these premises, the present study tested the hypothesis that the same gene x environment (G×E) interactions may predispose to PSU in college students and analyzed whether the influence of these biosocial interplays may follow a sex-dimorphic pattern.

## Methods

### Participants

Participants were 470 students (239 females and 231 males; see **Table 1**) enrolled in undergraduate psychology courses at a large Midwestern university. Recruitment was based on SONA, an online system that allows students to electronically sign up to participate in active studies at the university. Most students (71.1%) identified as Caucasians, attended the first year of college (61.1%) and reported that their parents had a higher educational level than high school (80.9% of fathers and 79.7% of mothers).

**Table 1:** Participant demographics and descriptive statistics.

	Overall Sample (n = 470)	Males (n = 231)	Females (n = 239)
*M* (*SD*) Age	18.95 (1.19)	19.14 (1.25)	18.76 (1.10)
Year in school			
% 1^st^ year student	61.1	55.8	66.1
% 2^nd^ year student	27.4	29.4	25.5
% 3^rd^ year student	8.9	11.7	6.3
% 4^th^ year student	1.9	2.6	1.3
% 5^th^ year or more student	0.7	0.5	0.8
Race/Ethnicity			
% Caucasian	71.1	72.7	69.5
% African American	3.6	3.0	4.2
% Hispanic/Latino	6.2	4.8	7.5
% Native American	1.3	.9	1.7
% Asian	10.6	10.4	10.9
% Mixed or other	7.2	8.2	6.2
Medical History			
% Psychological Disorder	13.2	10.4	15.9
% Current Illness/Injury	3.4	3.5	3.3
% Currently Medications	43.4	25.1	61.1
Parental Education at birth			
% Fathers greater than high school	80.9	81.0	78.4
% Mothers greater than high school	79.7	83.8	78.2

### Procedures

All study procedures were approved by the researchers' Institutional Review Board. All participants were instructed to abstain from eating for 1 h before the study, and refrain from the use of any drug (including prescription medicines and caffeinated beverages) for at least 3 h before the study. Upon arrival, they were given a complete summary of the study and provided informed consent. Subsequently, participants rinsed their mouth with water and, ten minutes later, were instructed to give 2 ml of saliva in a tube for genetic analyses. Then, they provided demographic information, including their age and race/ethnicity, and completed a Qualtrics online survey in about 1 h. At the end of the study, participants were compensated with a $5 debit card for the saliva sample and 3 SONA credits for the survey. To keep the identity of participants anonymous, survey responses and saliva samples were assigned a unique ID without any identifying information.

### Questionnaires

The survey included the following questionnaires:

1. The *Child Trauma Questionnaire* (CTQ), a standardized self-report instrument for the retrospective assessment of trauma exposure during childhood (Bernstein and Fink, 1998). The CTQ consists of 5 subscales of trauma (physical abuse, emotional abuse, sexual abuse, physical neglect, and emotional neglect) with multiple items based on a 5-point Likert scale format. Mean scores for each subscale, as well as an overall child maltreatment score, were calculated. The physical neglect subscale yielded the lowest reliability coefficient (α = 0.56) in the current sample; internal consistencies for the remaining four subscales were good (with all *α*'s > 0.81);
2. A *substance use questionnaire*. based on three items from the Center for Substance Abuse Prevention (CSAP) Student Survey (Pentz et al., 1989), a self-report instrument assessing lifetime tobacco (i.e., “Have you ever smoked a cigarette, even just a few puffs, or used chewing tobacco, snuff, or dip), alcohol (i.e., “Have you ever had a drink of alcohol?”), and cannabis use (i.e., “Have you ever tried marijuana?”). The number of substances used by each participant was calculated (ranging from 0 to 3).

### *MAOA uVNTR* Variants Genotyping

DNA extracted and *MAOA-uVNTR* genotyping were performed as previously described (Fite et al., 2018). All laboratory procedures were carried out by personnel blind to the demographic and psychological characteristics of the subject (other than gender). All genotype data of participants are shown in **Table 2**. Given that the *MAOA* gene is located on the X chromosome, males were designated as either low-activity (*MAOA-L*) or high-activity (*MAOA-H*) hemizygous, depending on the number of repeats of their allelic variant (2 and 3 vs 3.5 and 4, respectively). Conversely, females were either homozygous for either allele (*MAOA-LL* or *MAOA-HH*) or heterozygous carriers (*MAOA-LH*). In line with previous studies on *MAOA* (Byrd and Manuck, 2014), carriers of 5-repeat *uVNTR* alleles were excluded from the analyses, as the actual functional significance of this variant remains controversial (Sabol et al., 1998; Deckert et al., 1998). To allow for comparability between males and females, *MAOA-LL* and *MAOA-LH* female participants were combined (n = 165), in agreement with previous functional studies on sex-dimorphic effects of *MAOA uVNTR* variants (Fan et al., 2003; Meyer-Lindenberg et al., 2006; Frazzetto et al., 2007; Buckholtz et al., 2008; Dannlowski et al., 2009) The validity of this approach was confirmed by analyzing the interactions of *MAOA* genotype variants (MAOA-LL, MAOA-HH, and MAOA-LH) and maltreatment types in female participants. The results of these analyses indicated that *MAOA-LH* genotype operated consistent with the *MAOA-LL* genotype in its interaction with maltreatment types to predict PSU. All genotypic and phenotypic data are presented as **Supplementary Materials**.

**Table 2:** Genotypic data of all participants. Genotypes containing 5-repeat variants were not included in either MAOA low-activity (MAOA-L) or high-activity (MAOA-H) allele groups. For more details, see text.

	MALES		
	Number of repeats	Number	Percentage
*MAOA-L*	2	1	0.43%
	3	93	39.57%
*MAOA-H*	3.5	10	4.26%
	4	127	54.04%
Excluded genotypes	5	4	1.70%
	FEMALES		
	Number of repeats	Number	Percentage
*MAOA-LL*	2-2	1	0.41%
	2-3	1	0.41%
	3-3	42	17.28%
*MAOA-LH*	2-3.5	0	0%
	2-4	1	0.41%
3-3.5	2	0.82%
	3-4	118	48.56%
*MAOA-HH*	3.5-3.5	0	0%
	3.5-4	4	1.65%
	4-4	70	28.81%
Excluded genotypes	2-5	0	0%
	3-5	0	0%
	3.5-5	0	0%
	4-5	4	1.65%

### Data Analysis

Of the original 500 students recruited for the study, M*AOA* genotyping could not be performed for 11 participants, while 11 participants were missing CTQ and/or substance use data. We further excluded 8 participants (4 males and 4 females) carrying 5-repeat *uVNTR* alleles. Based on power tables (Aiken and West, 1991), it was determined that the current sample had adequate power (α = 0.80) to detect moderate to large, but not small, *MAOA* × maltreatment interaction effects for males and females. No differences in sex or age (*ps* > 0.48) or in child maltreatment scores (*p*s > 0.16) were found in the comparison between the participants included in and excluded from the analyses. Multiple regression models were used to evaluate proposed associations. Substance use count was the dependent variable in each model, with sex, *MAOA* variant, and maltreatment types included as independent variables. All five maltreatment types were included in each model to evaluate unique associations. Three-way interactions (e.g., sex × *MAOA* variant × maltreatment type) were then evaluated one at a time to determine if child maltreatment-MAOA interactive effects depended on sex. All independent variables were mean centered prior to analyses to aid in the interpretation of interaction effects. Statistically significant interactions were probed based on sex (male vs. female) and for *MAOA* variants to determine the nature of the interactions, consistent with standard procedures (Aiken and West, 1991).

## Results

Approximately 11.5% of the sample had not used any substance, 28.9% of the sample had used one substance, 23.2% had used two substances, and 36.4% of the sample had used three substances. Based on the clinical cutoff scores recommended by Bernstein and Fink (1998), ~46.5% of the sample reported at least low levels of one or more maltreatment types. This percentage is consistent with previous data on undergraduate, emerging adult samples (Reichert and Flannery-Schroeder, 2014).

Regression analyses indicated a significant three-way interaction when examining any experience of maltreatment (B = 1.36, *p* = 0.00; see **Table 3**). Additionally, a significant three -way interaction was found for physical abuse (B = 1.37, *p* = 0.00) as well as emotional abuse (B = 0.58, *p* = 0.04). However, no significant three -way interactions were found for any other child maltreatment type: physical neglect (B = 0.54, *p* = 0.26), emotional neglect (B = 0.40, *p* = 0.15), or sexual abuse (B = 0.43, *p* = 0.39). Additionally, no significant two-way interactions between maltreatment variables and *MAOA* alleles were evident (*p*s > 0.12).

**Table 3:** Three-way interaction regression analyses. Significant results are indicated in bold.

	SU Count	
	B	*p*
Sexual Abuse	0.43	0.39
Emotional Neglect	0.40	0.15
Physical Abuse	1.37	**0.00**
Emotional Abuse	0.58	**0.04**
Physical Neglect	0.54	0.26
Any Maltreatment	1.36	**0.00**

**Figure 1:**
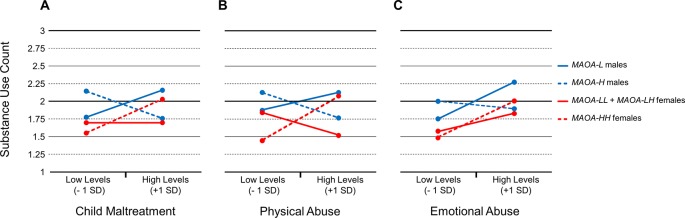
Associations between child maltreatment types and substance use count for male and female carriers of *MAOA uVNTR* variants. **(A)** Overall associations with child maltreatment scores. **(B)** Associations with physical abuse scores. **(C)** Associations with emotional abuse scores.

The statistically significant three -way interactions with any maltreatment type, physical abuse, and emotional abuse were further evaluated by conducting tests of the simple slopes (**Table 4**). Specifically, the models were conditioned at *MAOA-H* and *MAOA-L* for both males and females to determine the patterns of associations. For *MAOA-L* males, there was a marginally statistically trend for any maltreatment type (B = 0.42, *p* = 0.08) (**Figure 1A**) and statistically significant effect for and emotional abuse (B = 0.38, *p* = 0.03) (**Figure 1C**) to be positively associated with the number of substances used. However, an association between physical abuse and number of substances used was not found (B = 0.26, *p* = 0.17) (**Figure 1B**). For *MAOA-H* males, any maltreatment type was marginally statistically negatively associated (B = -0.42, *p* = 0.07) (**Figure 1A**) and physical abuse was statistically negatively associated (B = -0.33, *p* = 0.03) with the number of substances used (**Figure 1B**). Emotional abuse (B = -0.05, *p* = 0.77) was statistically unrelated to number of substances used for *MAOA-H* males (**Figure 1C**).

**Table 4:** Simple-slope analyses of three-way interactions. SE, standard error. *p < 0.05; ^+^p < 0.09.

	Males				Females			
	*MAOA – L*		*MAOA – H*		*MAOA – LL+ MAOA-LH*		*MAOA – HH*	
	B	SE	B	SE	B	SE	B	SE
Any Maltreatment	0.42^+^	0.24	–0.42^+^	0.23	0.00	0.16	0.52*	0.26
Physical Abuse	0.26	0.19	–0.33*	0.15	–0.25	0.19	0.54*	0.24
Emotional Abuse	0.38*	0.17	–0.05	0.17	0.19	0.14	0.34*	0.17

In contrast, for female carriers of low-activity MAOA variants (*MAOA-LL* and *MAOA-LH),* there was no association evident between any maltreatment type (B = 0.00, *p* = 0.99) (**Figure 1A**), physical abuse (B = -0.25, *p* = 0.18) (**Figure 1B**), or emotional abuse (B = 0.19, *p =* 0.17) (**Figure 1C**) and number of substances used. For homozygous *MAOA-H* females, there was a statistically significant positive association between any maltreatment type (B = 0.52, *p* = 0.04) (**Figure 1A**), and physical abuse (B = 0.54, *p* = 0.03) (**Figure 1B**), and emotional abuse (B = 0.34, *p* = 0.04) (**Figure 1C**) and number of substances used.

## Discussion

The results of the current study showed that, in a sample of students enrolled in a large Midwestern university, PSU was predicted by the interaction of *MAOA uVNTR* allelic variants, sex, and specific child maltreatment types. The highest number of substances used was found in *MAOA-L* male and *MAOA-HH* female carriers with a history of emotional abuse (as well as physical abuse in women). To our knowledge, this is the first report documenting a key role of *MAOA* as a mediator of child maltreatment with respect to PSU. While previous studies have shown the importance of G×E interactions in PSU (Vaughn et al., 2009; Rende, 2011), the specific genetic factors implicated in such biosocial interplays remain mostly elusive; if confirmed by future studies, our results may point to *MAOA* as a key molecular basis for PSU.

The present findings extend our previous report of sex-dimorphic influences of G×E interactions in the lifetime use of tobacco (Fite et al., 2018) among college students. Furthermore, these results are consistent with previous evidence indicating sex differences in the interactive influence of these G×E interactions with respect to antisocial conduct (Nikulina et al., 2012; Stogner and Gibson, 2013; Byrd and Manuck, 2014; Harro and Oreland, 2016) and alcohol use (Nilsson et al., 2011). The interaction of *MAOA* alleles and child maltreatment can be interpreted from the perspective of the diathesis-stress model, which postulates that the predisposition to specific neurobehavioral deficits is the result of a synergistic combination of genetic and environmental untoward factors (Zuckerman, 1999). Another alternative interpretation follows the differential susceptibility hypothesis, which posits that specific genetic variables may sensitize to both the positive and the negative influence of early experiences (Ellis et al., 2011). This possibility is partially supported by Belsky and colleagues (Belsky et al., 2009; Belsky and Beaver, 2011), who have conceptualized that *MAOA* variants may act as plasticity factors in the predisposition to substance use and other psychopathological conditions.

In line with previous data (Armour et al., 2014), the current results highlight the importance of examining specific maltreatment types in relation to PSU. Our findings suggest that, although physical abuse and emotional abuse interact with *MAOA* variants to predict PSU even when statistically controlling for the other maltreatment types, no interaction effects were found for sexual abuse, physical neglect, or emotional neglect. This evidence is partially consistent with a previous study by Nikulina and colleagues (2012) suggesting that *MAOA* does not serve as a protective or risk factor for substance use outcomes among individuals who have experienced childhood sexual abuse. However, in contrast with our results, the results of that investigation showed that alcohol use was not predicted by the interaction of *MAOA* with either physical abuse or neglect. Given that the participants of that study ranged between 31 and 51 years of age, it is possible that the discrepancy with those results may reflect age differences; accordingly, the moderating effect of *MAOA* on child maltreatment and negative outcomes has been hypothesized to be age-dependent (Huizinga et al., 2006). Alternatively, these divergent findings may result from other differences between our studies, including the substance use outcomes (i.e., PSU vs alcohol abuse) and measurement of child maltreatment (i.e., self-report vs official records). Nevertheless, research shows that experiences of child maltreatment are associated with decreased propensity for reward selection, which could be due to lower reward sensitivity (Guyer et al., 2006). In turn, this dual risk might increase the risk of PSU. Thus, child maltreatment types, physical abuse and emotional abuse may be more saliently associated with blunted reward sensitivity.

The existence of sex-dimorphic G×E interactions involving *MAOA uVNTR* alleles has been attested in other psychopathological states. For example, male carriers of *MAOA-L* alleles with a history of child maltreatment have a significantly higher risk of antisocial, aggressive, and violent behavior (Caspi et al., 2002; Kim-Cohen et al., 2006; Beaver et al., 2010; Aslund et al., 2011; Fergusson et al., 2011; Fergusson et al., 2012; Byrd and Manuck, 2014; Godar et al., 2016). Notably, the same G×E interaction has been reproduced in mouse models, further supporting the biological nature of this biosocial interplay (Godar et al., 2019). Conversely, female carriers of *MAOA-H* alleles with a positive history for early-life adversity display a higher proclivity for antisocial and violent responses (Sjöberg et al., 2007; McGrath et al., 2012; Verhoeven et al., 2012). It has been hypothesized that this effect may reflect the enhancement of emotional reactivity during adolescence (Byrd et al., 2018). Furthermore, these effects may reflect sex- and genotype-specific differences in the effects of MAOA on monoamine metabolism (Jönsson et al., 2000; Aklillu et al., 2009). Notably, aggression and delinquency have been extensively linked to PSU, particularly in boys (McCormick and Smith, 1995; Mason and Windle, 2002; Martinotti et al., 2009). This concurrence strongly suggests that the G×E interaction of *MAOA* genotype and child maltreatment may predispose to a broad set of externalizing responses, ranging from antisocial personality to PSU propensity. In line with this interpretation, neuroimaging studies have pointed to *MAOA* as a key molecule to influence the function of the anterior cingulate cortex (ACC) (Passamonti et al., 2008). This region plays a major role in the regulation of self-regulation (Posner et al., 2007), the key domain implicated in the ontogeny of antisocial behavior (Gardner et al., 2008; Trentacosta and Shaw, 2009; Gillespie et al., 2018), as well as in the role of G×E interactions in PSU (Vaughn et al., 2009). The effects of *MAOA* on ACC activation patterns are sex-dimorphic; specifically, *MAOA-L* male and *MAOA-H* female carriers with a history of early stress display impairments in the activation of the ACC in response inhibition (Holz et al., 2016), a process directly related to self-regulation (Posner and Rothbart, 1998; Blair and Ursache, 2011; Hofmann et al., 2012). It should be noted that functional deficits of the ACC are associated with a reduction in inhibitory control (Bush et al., 2000; Chan et al., 2011), as well as a facilitation of ventral striatal responses to incentive stimuli, which in turn increases drug use propensity (Holmes et al., 2016; Koyama et al., 2017). Notably, these deficits may be particularly overt in young individuals (and therefore highly relevant in the age range of college students), due to their incomplete myelination of the ACC as well as the development of the dopaminergic system, which further exacerbates their proclivity to engage in impulsive and risky actions and heightens their reward sensitivity (Casey et al., 2008; Steinberg, 2008). At least in females, the presence of *MAOA-H* alleles may further reduce dopamine levels, ultimately promoting the ontogeny of reward deficiency syndrome (Blum, 2017; Blum et al., 2018). From this perspective, these results suggest that the interaction of *MAOA-L* alleles in males and *MAOA-H* in females and early-life maltreatment may interfere with the development of inhibitory control in emerging adulthood, ultimately increasing PSU risk.

Several limitations of this study should be acknowledged. First, our analyses focused exclusively on *MAOA* polymorphisms, yet several studies point to the importance of many other genes in the vulnerability to PSU, such as those encoding for dopamine receptor 2 and 4 as well as dopamine and serotonin transporters (Blum et al., 2010); further studies are needed to evaluate the potential interaction of child maltreatment with these vulnerability factors. Second, although rich literature has documented that *MAOA* variants interact with childhood maltreatment to increase the propensity for externalizing behaviors, our findings need to be replicated in larger samples from multiple colleges and with less skewed ethnic distribution. Indeed, our sample comprised of predominantly Caucasian youth, which may limit the generalizability of current results. Second, this study relied solely on self-reports of constructs, with a low internal consistency associated with our measure of physical neglect. Future research examining associations in other samples (e.g., clinical and criminal) using multiple, psychometrically sound assessments of constructs would be useful for establishing generalizability of findings. Finally, our research combined two- and three-repeat variant carriers in the *MAOA-L* group; however, previous studies, however, have shown that, in males, two-repeat alleles resulted in much lower levels of promoter activity as well as stronger phenotypic effects than the three-repeat genotype (Sabol et al., 1998; Guo et al., 2008). Notably, two-repeat variants have shown to increase antisocial phenotypes, including the propensity to engage in particularly violent conduct (such as shooting and stabbing), in African-American males (Beaver et al., 2013; Beaver et al., 2014). Unfortunately, given that only one male participant was found to carry the two-repeat alleles, our analyses were not sufficiently powered to differentiate across specific genotypes; however, future studies will be needed to verify whether specific differences may be identified with respect to the interaction of specific variants with early maltreatment.

Despite these limitations, the current study contributes to the growing literature indicating sex differences in genetic risk of MAOA in addition to the importance of the interactive influences of genetic and environmental risk for PSU. Further, findings indicate the importance of evaluating specific maltreatment types to better understand *MAOA* and maltreatment interactive risks for substance use.

## Data Availability Statement

All datasets generated for this study are included in the article/**Supplementary Material**.

## Ethics Statement

The studies involving human participants were reviewed and approved by IRB University of Kansas. The patients/participants provided their written informed consent to participate in this study.

## Author Contributions

PF conceptualized the project, supervised data collection and analysis, and drafted the first draft of the manuscript. SB helped with data collection and analysis and helped draft the first version of the manuscript. WH performed genotyping analyses and drafted part of the method sections. AM and MGB contributed to the conceptualization of the study and reviewed genotyping data. MB conceptualized the project, reviewed data analysis, and wrote the final version of the manuscript. All authors have read and approved the final version of the manuscript.

## Conflict of Interest

The authors declare that the research was conducted in the absence of any commercial or financial relationships that could be construed as a potential conflict of interest.
